# Psychological and Mental Health Support for Vietnamese University Students in Economics Majors: Approaches and Needs Assessment

**DOI:** 10.3390/ijerph23020232

**Published:** 2026-02-11

**Authors:** Ngoc Bich Luu, Hà Thanh Nguyễn, Ngoc Bao Nguyen, Son Hong Dang, Hoa Quynh Nguyen

**Affiliations:** 1 Office of the National Council for Education and Human Resource Development, Hanoi 100000, Vietnam; 2 Faculty of Development Economics, National Economics University, Hanoi 100000, Vietnam; hant@neu.edu.vn; 3 Advanced Education Program (AEP), Intake 64, National Economics University, Hanoi 100000, Vietnam; ngocnb.kdqt@gmail.com; 4 Faculty of Economics and Human Resource Management, National Economics University, Hanoi 100000, Vietnam; sondh@neu.edu.vn (S.H.D.); hoanguyen@neu.edu.vn (H.Q.N.)

**Keywords:** psychological and mental health support, needs assessment, Vietnamese university students

## Abstract

**Highlights:**

**Public health relevance—How does this work relate to a public health issue?**

- This study addresses a pressing public health concern in Vietnam: the high prevalence of psychological distress among university students, driven by academic workload, financial instability, family expectations, and career uncertainty.
- It demonstrates that unmet needs for professional and confidential psychological support within universities represent a significant gap in the current mental health response system.

**Public health significance—Why is this work of significance to public health?**

- Using a mixed-methods cross-sectional design with 701 respondents from multiple economics-oriented universities across three regions, the research provides robust and discipline-specific evidence on students’ support needs.
- The findings enhance international and national discoverability of Vietnamese student mental health patterns and offer reliable material that can be cited by other scholars in educational and community health fields.

**Public health implications—What are the key implications or messages for practitioners, policy makers and/or researchers in public health?**

- Results call for institutionalized periodic screening, expansion of individual counseling services, and the development of low-threshold digital counseling platforms aligned with youth habits.
- Integrating career guidance with academic skills mentoring and emotional regulation interventions is identified as the key message for practitioners and policy makers to prevent escalation to depression, social withdrawal, and reduced learning motivation.

**Abstract:**

The mental health of students in university has become an increasingly pressing concern due to rising academic pressure, career uncertainty, and major life transitions. Identifying students’ psychological support needs requires an understanding of the challenges they face, as well as their expectations regarding support forms, intervention methods, and service providers. This study employed a mixed-methods cross-sectional design, combining large-scale questionnaire surveys (701 respondents) with qualitative interviews to assess the mental health status and psychological support needs of students at economics universities in Vietnam. The findings reveal that students commonly experience negative emotional states, particularly anxiety related to academic workload, financial instability, personal health, and future career orientation. A proportion of students reported depressive symptoms such as persistent sadness, prolonged stress, and physiological disturbances including insomnia and disordered eating. While severe behavioral disorders are uncommon, signs of declining academic motivation, social withdrawal, and weakened interactions with lecturers are evident. Students express a strong demand for mental health support, especially in career guidance, learning strategies, emotional regulation, and interpersonal problem-solving. Individual, professional, confidential counseling services are the most preferred forms of support, highlighting the need for a comprehensive mental health and psychological support system tailored to the context of Vietnamese universities.

## 1. Introduction

International studies over the past two decades indicate that the mental health (MH) of university students is a global concern and has increasingly attracted scholarly and educational attention. Ibrahim et al. [1], in a systematic review of 24 studies conducted between 1990 and 2010, reported that up to 30.6% of university students worldwide experienced depressive symptoms—a significantly higher rate than that observed in the general population. This finding was reinforced by Sarokhani et al. [2], whose analysis of 35 studies in Iran showed an average depression prevalence of 33% among students, with higher rates among females and unmarried students. Similarly, Dell’ Osso et al. [3] found that 29.7% of students at the University of Pisa (Italy) exhibited symptoms of social anxiety that undermined self-esteem and academic performance.

Beyond depression and anxiety, numerous studies have highlighted the relationship between psychosocial factors and student mental health. Kabir [4] identified anxiety, depression, obsessive–compulsive disorder, and eating disorders as common issues among students, emphasizing the role of psychological support, physical activity, and nutrition in improving mental well-being. Reis and Matos [5], in a study conducted in Portugal, reported that students frequently experienced anxiety in five key domains: academics, time management, finances, family circumstances, and personal health. Jefferies et al. [6] further noted that global social anxiety prevalence among young adults could reach 36%, with contributing factors including academic pressure, social media exposure, and societal expectations.

Recent studies have continued to clarify how school environments and social conditions influence student mental health. Jiang et al. [7] found that 24.9% of Chinese university students exhibited anxiety and 16.3% displayed depressive symptoms, mostly attributed to academic pressure, lack of social support, and career instability. Yu Jin et al. [8] reported that 38% of students experienced eating disorders associated with body-image anxiety. Bantjes et al. [9], in South Africa, demonstrated that suicidal behaviors and ideation were strongly linked to academic stress, social isolation, and insufficient access to professional support.

With respect to interventions, numerous studies have proposed need-based mental health support models for higher education institutions. Uchida and Hashimoto [10] demonstrated that reciprocal social support plays a crucial role in maintaining positive mental health among students. Katauke [11] highlighted the effectiveness of integrated psychological counseling models incorporating self-awareness training and emotional management skills in preventing academic depression. Following this evidence, international organizations such as UNICEF [12] and WHO [13] have recommended embedding mental health programs within educational systems, emphasizing three foundational components: raising awareness, building professional support capacity, and developing school-based counseling networks.

In Vietnam, rapid economic growth, scientific advancement, technological innovation, and the expansion of social media have brought significant benefits, yet they have also generated substantial negative consequences for both physical and, more critically, mental health. Psychological and mental health issues have become major concerns across various sectors, with education considered one of the most affected environments. A nationwide survey by VNU, Hanoi [14], reported that 8% to 29% of adolescents and university students showed signs of mental distress; 26.3% exhibited depressive symptoms, 6.3% reported suicidal ideation, and 5.8% had attempted suicide. UNICEF [12] further identified academic pressure, family expectations, and the social environment as major risk factors influencing school-based mental health.

Several recent studies have examined the prevalence and determinants of student mental health issues in Vietnam. Ly and Vo [15] found that 96.2% of students displayed symptoms of depression, anxiety, or stress, with over 30% experiencing severe levels. Risk factors included heavy academic workloads, exam pressure, financial difficulties, and interpersonal conflicts. Quynh Chi, N. et al. [16] emphasized the urgent need to establish school-based psychological support models, particularly given that students rarely seek counseling services proactively.

Another line of research has explored protective factors and support models. Nguyen Thi Mai Huong [17] noted that positive coping skills, emotional self-regulation, and social connectedness play essential roles in sustaining student mental well-being. Meanwhile, Tran Thi Thu Ha et al. [18] proposed a three-tiered support model: (i) universal psychological skills education for all students; (ii) group-based counseling for those experiencing stress; and (iii) specialized intervention for students with severe psychological disorders. However, these models have been tested primarily on small scales and lack nationwide implementation guidelines.

A noteworthy gap in the Vietnamese literature is that most studies examine student mental health in general, with limited attention to specific academic disciplines. In particular, despite economics being the largest academic cluster in Vietnam’s higher education system [19], there has been little research that systematically analyzes the characteristics, support needs, or appropriate intervention models tailored to students in this field. Yet empirical evidence suggests that economics students frequently face high academic pressure, intense career competition, limited opportunities for practical training, and scarcity of experiential learning, leading to prolonged stress and anxiety.

Overall, both international and Vietnamese studies demonstrate that university student mental health is a widely recognized issue, with substantial evidence pointing to high levels of depression, anxiety, and stress. However, international studies tend to focus more on symptom descriptions and risk factors rather than specific support needs, while intervention models vary considerably across cultural and national contexts. In Vietnam, most existing studies remain at the level of general assessment or broad recommendations. A significant research gap persists regarding the specific mental health support needs of economics students—an academic group characterized by unique academic pressures, career orientations, and social environments. Literature review showed that many studies have employed longitudinal data. Such data demonstrate that issues such as mental health are dynamic processes and that significant differences exist across different population groups.

At the university level, Vietnamese students encounter multiple pressures, including navigating a highly competitive admissions system, adapting to new learning environments, living away from their families, managing heavy academic workloads, and meeting societal expectations regarding academic performance and career pathways. These factors contribute to heightened risks of psychological disorders and mental health challenges among students. Some studies reveal that students may experience many psychological difficulties, such as anxiety disorders (fear, worry), depression (sadness, emptiness, stress), behavioral disorders (impulsivity, aggression), psychotic disorders (hallucinations, delusions), physiological disturbances (eating disorders, sleep disorders, low self-worth and challenges in career orientation, problems adapting to academic environments, difficulties in learning processes, family relationship conflicts, challenges in relationships within the university, and broader social relationship problems, loss of confidence, declining academic interest, acceptance of substance use (alcohol, tobacco, drugs), social withdrawal or avoidance, destructive interpersonal behaviors, and self-harm (suicidal or self-injurious acts). They are grouped into several clusters: (1) *Negative*/*pathological emotional states*; (2) *Cognitive difficulties*; (3) *Environmental and social adaptation difficulties*; (4) *Maladaptive behaviors and attitudes* [20]. Numerous studies also emphasize the importance of early identification of psychological support needs and implementing timely interventions starting from students’ first year of enrollment [21].

Within the broader university population, economics majors account for a substantial and increasing proportion of students in recent years. Data from the Ministry of Education and Training (2024) show that the fields of *Business and Management* had the highest number of applications in three consecutive admission cycles: 32.77% (2022), 24.54% (2023), and 27.42% (2024). Although Vietnam offers nearly 400 majors across 24 fields, economics-related programs consistently rank among the most competitive and most preferred.

Economics programs are often perceived as “easier” in terms of academic structure compared with engineering disciplines, while offering promising career prospects and higher income potential than fields in the social sciences and humanities. However, this perception unintentionally generates a dual pressure for students: the need to meet high expectations from families and society, and the requirement to excel in a learning environment that demands both quantitative and qualitative competencies. Many students experience an “academic shock” when transitioning from exam-oriented learning in high school to university-level study that emphasizes independent thinking and self-directed research. Additionally, economics programs typically lack laboratory-based or fieldwork components, increasing students’ pressure to independently build professional skills, secure internships, and compete for jobs after graduation [22].

Within this context, economics students are particularly vulnerable to anxiety, stress, declining academic motivation, and career-related crises. Despite this, there remains a lack of specialized research assessing the psychological difficulties and mental health support needs of this specific student group. Moreover, empirical evidence on the mental health support needs of economics students in Vietnam is limited, hindering the development of targeted and context-appropriate intervention models.

This study aims to identify the psychological difficulties experienced by economics students in Vietnam and examine their mental health support needs. The findings will contribute to proposing appropriate interventions from families, universities, and society through a need-based approach, with the overarching goal of enhancing the effectiveness of mental health care and support for Vietnam university students.

## 2. Materials and Methods

### 2.1. Approaches and Theoretical Framework for Support Need Measurement

Mental health (MH) and psychological problems have become major concerns in higher education, reflecting a complex interplay of biological, psychological, and social factors that affect students’ holistic development. Psychological problems, according to Selye [23], include maladaptive states that impair emotions, behavior, and adaptive capacity, such as stress, anxiety, depression, and sleep disturbances. Beck [24] argued that when psychological difficulties exceed an individual’s capacity for self-regulation, they may evolve into psychiatric disorders that require professional intervention. “Psychological difficulties” denote a mismatch between an individual’s psychological characteristics and their situational demands, manifesting in maladaptive cognitions, attitudes, and behaviors [25,26]. Studies by Mirowsky & Ross [27] and Kessler et al. [28] indicate that psychological difficulties are often associated with an imbalance between personal needs and coping capacity, negatively affecting quality of life.

The concept of “mental health” is approached from multiple disciplines, including psychiatry, psychology, and social work. The World Health Organization [29,30] defines mental health as a state of well-being in which an individual realizes their potential, can cope with normal stresses of life, can work productively, and can contribute to their community. John-Langba J. et al. [31] reaffirm that mental health is a complex construct reflecting the integration of biological, psychological, and social functioning. Dodd, A. B. et al. [32] emphasize that mental health is not merely the absence of pathology but the capacity to maintain internal balance, recover from adversity, and lead a fulfilling life.

In the context of higher education, individuals aged 18–24 are considered the most psychologically vulnerable. This period involves identity formation, self-definition, career orientation, and social positioning. Kessler et al. [33] report that more than half of psychiatric disorders have their onset during youth, increasing the risk of school dropout, unemployment, and difficulties in social integration. Wang [34] highlights the importance of mental health education within universities as an effective preventive measure to limit early onset disorders. According to OECD [35] and PISA [36], student mental health comprises four interrelated dimensions—psychological, social, cognitive, and physical—which interact reciprocally during the adaptation and development process.

The psychological dimension encompasses self-awareness, motivation, self-esteem, and emotional resilience [9,37]. The social dimension reflects the quality of interpersonal relationships and the degree of connectedness to the community [38]. The cognitive dimension refers to critical thinking ability, problem-solving skills, and mastery of knowledge, whereas the physical dimension is associated with physiological health, sleep, nutrition, and physical activity [39].

The literature indicates that psychological difficulties inevitably give rise to the need for psychological and mental health support. Effective psychological and mental health interventions for students must therefore be grounded in their actual support needs. According to Lovibond and Lovibond cited in Qi Jiang [21], students may experience 12 types of psychological difficulties, grouped into four clusters: (1) *Negative*/*pathological emotional states*: anxiety disorders (fear, worry), depression (sadness, emptiness, stress), behavioral disorders (impulsivity, aggression), psychotic disorders (hallucinations, delusions), and physiological disturbances (eating disorders, sleep disorders); (2) *Cognitive difficulties*: low self-worth and challenges in career orientation; (3) *Environmental and social adaptation difficulties*: problems adapting to academic environments, difficulties in learning processes, family relationship conflicts, challenges in relationships within the university, and broader social relationship problems; (4) *Maladaptive behaviors and attitudes*: loss of confidence, declining academic interest, acceptance of substance use (alcohol, tobacco, drugs), social withdrawal or avoidance, destructive interpersonal behaviors, and self-harm (suicidal or self-injurious acts).

The concept of “need” is central to psychological research, reflecting what individuals perceive as lacking or requiring fulfilment to achieve physical, mental, and social balance. In psychology, needs are viewed as multilayered constructs that represent internal motivations and the dynamic interaction between individuals and their social environment. The *APA Dictionary of Psychology* [37] defines psychological needs as essential non-biological conditions for mental well-being, arising from internal factors such as the desire for recognition or love, or from social interactions involving fairness, autonomy, or work satisfaction. Maslow’s [40] hierarchy of needs describes five tiers—from physiological needs to safety, belongingness, esteem, and self-actualization—representing the progression of personality development and behavioral motivation. Similarly, Deci and Ryan’s [37] theory of basic psychological needs identifies three core needs—autonomy, competence, and relatedness—as foundations for mental health and intrinsic motivation.

According to the World Health Organization [41], “mental health and psychosocial support” (MHPS) comprises multidisciplinary activities that address social, psychological, and psychiatric issues, implemented by professionals across multiple sectors. In higher education contexts, *support for students* includes both academic and non-academic measures designed to help learners adapt, build personal competencies, manage psychological difficulties, and achieve academic success [42].

The concept of *school-based psychological support* has been interpreted in various ways by Vietnamese scholars. Duc, T.T.M. [43] defines it as intervention activities aimed at fostering optimal development among students. Nguyen Thi Minh Hang [44], in contrast, views it as a system that applies psychological knowledge to practice, enabling learners to independently resolve their own issues in a positive manner. Rogers [2] and Neukrug [45] emphasize the role of school counseling as a collaborative, interactive process that supports individuals in developing self-awareness and coping capacity. In the Vietnamese context, Bui, T. X. M. [5] characterizes counseling as a professional process that helps individuals identify their emotions, thoughts, and behaviors, thereby enabling them to utilize their internal resources to address psychosocial problems.

Drawing from these perspectives, “students’ psychological support needs” may be broadly understood as their desire to access and participate in supporting and/or counseling activities that help them overcome difficulties in learning, social relationships, emotional well-being, and career orientation. These needs manifest through students’ emotions, behaviors, and cognitions when they experience challenges or lack adaptive skills. Addressing such needs requires not only professional services but also coordinated efforts among universities, families, and broader society to create an environment that promotes healthy mental development.

Within Vietnamese higher education, recent studies indicate a growing demand for psychological support among students [46,47,48]. Students’ key areas of concern include academic difficulties, social adaptation, management of negative emotions, career guidance, and personal development. Issues such as anxiety, depression, exam pressure, and interpersonal conflicts not only affect academic performance but also undermine students’ motivation and self-regulation abilities [49,50]. Thus, identifying and responding to psychological support needs is crucial for enhancing student mental health and improving the overall quality of higher education.

ACT Alliance & Church of Sweden [37] outline four principles for implementing psychological and mental health support: ensuring safety; soothing negative emotions; enhancing individual and community values; and fostering social connectedness. These principles are highly relevant to the context of the present study.

Regarding support methods, the *Demand–Control Model* [42,51] is considered for application in this research. This model is widely used in mental health and psychological support interventions for young populations and encompasses four key components: assessing needs, providing support, increasing motivation in life or academic pursuits, and reducing risk factors that contribute to psychological and mental health difficulties during the university years. The present study focuses specifically on the need-assessment component.

**Figure 1:**
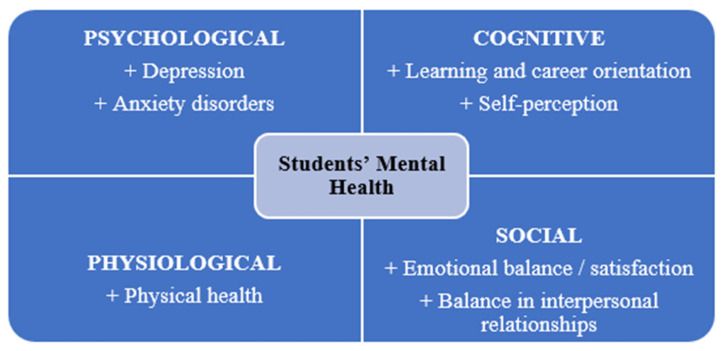
Dimensions of Students’ Mental Health [39,42].

Based on the literature review and drawing on the works of Rudnik, A. et al. [52] and Waselewski, E. A., Waselewski, M. E., & Chang, T. [53] on psychological support needs, as well as Lovibond cited in Qi Jiang [21], Travia, R.M. et al. [54], OECD [35] and others on psychological difficulties and mental health needs, the conceptual framework for determining psychological and mental health support needs for university students is developed as illustrated in Figure 1.

### 2.2. Questionnaires for Quantitative Survey

**Figure 2:**
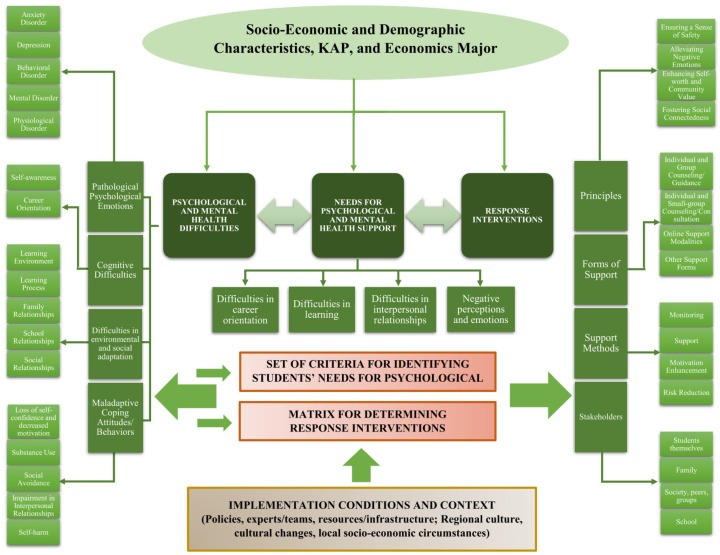
Research framework for identifying students’ psychological and mental health support needs.

The literature review suggests that psychological difficulties among university students give rise to corresponding needs for psychological and mental health support. Accordingly, interventions aimed at promoting students’ psychological well-being and mental health should be grounded in a clear understanding of these support needs. Existing studies indicate that psychological and mental health issues in university students can be conceptualized across four interrelated dimensions: (i) negative emotional states; (ii) maladaptive attitudes and behaviors; (iii) negative or pathological psychological manifestations; and (iv) cognitive difficulties. According to Lovibond and Lovibond as cited in Qi Jiang [21], university students may experience twelve manifestations of psychological difficulties, which are categorized into four domains: (1) negative emotions related to academic learning, (2) difficulties in adapting to the university environment, (3) negative psychological symptoms, and (4) difficulties in self-awareness. Corresponding to these four dimensions of students’ psychological and mental health problems, the authors reconstructed and reorganized sixteen indicators to capture these domains, including the following (Figure 2):

- *Negative emotional and pathological symptomatology*: Anxiety-related symptoms (e.g., fear, excessive worry); depressive symptoms (e.g., persistent sadness, emotional emptiness, chronic stress); behavioral dysregulation (e.g., impulsivity, aggression); psychotic-like symptoms (e.g., hallucinations, delusional thinking); and physiological disturbances associated with psychological distress (e.g., disordered eating and sleep disturbances).
- *Cognitive difficulties*: Impaired self-perception and self-worth, as well as difficulties in career orientation and decision-making.
- *Difficulties in environmental and social adjustment*: Challenges in adapting to the academic environment; learning-related difficulties; strained family relationships; difficulties in relationships within the university setting (e.g., with peers and lecturers); and challenges in broader social interactions.
- *Maladaptive behaviors and attitudes*: Reduced self-confidence and diminished academic engagement; acceptance or use of psychoactive substances (including alcohol, tobacco, and illicit drugs); social withdrawal and avoidance behaviors; actions that negatively affect interpersonal relationships; and self-harm behaviors, including suicidal ideation or non-suicidal self-injury.

The questionnaire was designed around four factor groups representing sixteen indicators of psychological and mental health difficulties. These indicators were developed through the adaptation and extension of variables derived from established psychological and mental health measurement instruments, including the GAD-7, DASS-21, SDQ, PHQ-9, RSES, and CDMSE. A standard five-point Likert scale was employed to measure the likelihood of students experiencing negative psychological and mental health symptoms. Response options of 1 and 2 indicated “very severe” and “severe”, respectively, whereas scores of 4 and 5 represented “not severe” and “not severe at all.” A score of 3 reflected an unclear state. The conceptual variables were operationalized into questionnaire items that were adapted to student behaviors and the cultural context of Vietnam, as illustrated in Table 1.

### 2.3. Data Collection

This study employed a mixed-methods cross-sectional design, combining large-scale questionnaire surveys with qualitative interviews. To collect data on the current state of students’ psychological and MH problems, as well as their support needs, the research team implemented a mixed-methods survey across economics-oriented universities in all three regions of Vietnam (North, Central, and South). The participating universities included: (1) National Economics University (NEU); (2) Foreign Trade University—Hanoi (FTU); (3) School of Economics—Vinh University (VinhUni); (4) School of Economics—University of Danang (DNU); (5) Hung Vuong University (HVU); (6) University of Economics Ho Chi Minh City (UEH); (7) University of Finance—Marketing Ho Chi Minh City (UFM).

A total of 701 self-administered questionnaires (via Google Forms) were collected. The questionnaire served as the core diagnostic tool for identifying students’ psychological and MH issues, developed from existing checklists assessing psychological difficulties and potential MH risks, and supplemented with sociodemographic questions to evaluate support needs. It should be acknowledged that although the survey sample was stratified by region and by institutional unit (universities) to enhance coverage and diversity, participants were selected using a non-probability convenience sampling approach based on random encounters. Consequently, the sample cannot be considered nationally representative. However, the stratified design and the inclusion of multiple universities across different regions help to capture a broad range of student experiences, allowing the findings to provide meaningful insights into prevailing mental health patterns and support needs among economic university students in Vietnam.

The quantitative sample size was determined based on the rule that factor analysis requires at least five times the number of observed variables [6,15]. As this study included 64 observed variables, the minimum required sample size was 320. However, because data collection was conducted online, the sample size was tripled to minimize sampling error. Ultimately, 903 students from economics-oriented universities participated in the survey. One exclusion criterion was institutional eligibility: participating institutions were required to have a sufficiently large student body with representation across multiple economics and business-related majors, rather than a single specialized program. Based on this criterion, responses from Hung Vuong University and the University of Finance–Marketing were excluded from the final analytical sample. After screening and quality assessment to ensure sample consistency and reliability, invalid responses were removed, leaving 701 valid questionnaires (students from 5 out of 7 universities) for statistical analysis and data processing. The detailed sample distribution is presented in the following Table 2.

The quantitative data collected via Google Forms were cleaned and subsequently analyzed using SPSS 22 (IBM. Corp., Armonk, NY, USA). The analysis included descriptive statistics, univariate and bivariate analyses, Exploratory Factor Analysis (EFA), Confirmatory Factor Analysis (CFA), correlation analysis, and verification of the research results (assess the reliability of the scale using Cronbach’s Alpha and statistical reliability).

Regarding sample characteristics, the majority of respondents were female, accounting for 76%, while male students represented only 24%. The gender distribution of the survey sample broadly reflects the actual gender composition of students in economics-related disciplines, where female students typically outnumber male students. This pattern is characteristic of the current disciplinary structure within Vietnamese higher education. By contrast, studies focusing on engineering or technical fields would be expected to report a higher proportion of male students than female students. Additionally, 16% came from wealthy households (families owning luxury assets such as cars and personal vehicles). Students from major cities accounted for 36% of the sample, 17% came from smaller urban areas, 35% from rural lowland regions, and 12% from mountainous rural areas. Overall, the proportion of students from urban settings was slightly higher than from rural areas (53% versus 47%). In terms of current living arrangements, most students reported either “living with family” (45.4%) or “living with friends in rented accommodation” (34.88%). Only 15.5% lived alone in rented housing, and 7.8% lived in dormitories. A small, negligible proportion lived with relatives or acquaintances.

For the qualitative component, 19 in-depth interviews were conducted. At each university where the questionnaire survey was administered, student participants were selected using convenience sampling; in-depth interviews were conducted with students who were randomly encountered on campus and consented to participate in the study. In addition, at each institution, one to two staff members from the student affairs office were selected using purposive sampling, as representatives of university leadership, and were interviewed to provide institutional perspectives.

The qualitative data were analyzed using qualitative content analysis, which was selected due to the structured nature of the interview protocol and the presence of predefined areas of investigation aligned with the study objectives. This approach is particularly appropriate for systematically interpreting textual data when analytic categories are informed by existing theory and research questions rather than being derived purely inductively [55,56].

The analytic procedure applied to the interview data is delineated as follows: Phase 1—All interview transcripts were systematically reviewed and independently coded by two members of the research team to enhance analytic rigor and minimize individual bias; Phase 2—The researchers conducted a comparative analysis of their coding outputs, addressing discrepancies through iterative discussion. This phase involved cross-validation of preliminary codes, critical examination of interpretive differences, and resolution of divergent perspectives to establish a coherent and reliable coding framework; Phase 3—The consolidated coding scheme was subsequently presented to the full research team for collective deliberation, enabling further refinement and ensuring methodological consistency across the study.

## 3. Results

### 3.1. Students’ Psychological Difficulties and Mental Health Problems

#### 3.1.1. Negative Psychological Emotions

The analysis shows that among the four dimensions of psychological difficulties, manifestations of negative psychological emotions appear with the highest frequency. These include frequent anxiety about managing time and organizing daily activities (such as scheduling classes, self-study, personal routines, and group activities); frequent worry about one’s financial situation (including tuition fees, living expenses, and unexpected costs); frequent concern about current family circumstances (such as the health of family members and household economic conditions); and persistent worry about personal health or physical appearance (physical health, body weight, attractiveness). All of these indicators recorded mean scores below 3 (Table 3). In this section, lower mean scores (below 3) indicate higher perceived severity of psychological difficulties, as defined by the 5-point Likert scale.

In addition, many students exhibited symptoms of depression, such as “frequently feeling bored, disappointed, or downhearted” and “frequently feeling stressed, pressured, and having difficulty initiating tasks” (mean score < 3) (Table 4).

Manifestations of psychotic disorders or behavioral disturbances are not yet severe among this group of students (mean score > 3) (Table 5). However, more concerning than these cognitive or behavioral symptoms are the physiological disturbances. Many students reported “frequently experiencing loss of appetite or overeating” and “frequently experiencing insomnia, lack of sleep, difficulty falling asleep, poor sleep quality, or oversleeping” (mean score < 3) (Table 5). These physiological disturbances negatively impact students’ physical health (e.g., becoming underweight or overweight), which in turn heightens their worries, as previously noted. Additionally, falling into such states implies a loss of control in maintaining a healthy study–life routine.

#### 3.1.2. Difficulties in Self-Perception

The survey results on students’ cognitive difficulties indicate two prominent categories: self-perception and career orientation. Among these, difficulties related to cognitive understanding and career orientation appear more pronounced than those related to self-perception alone. Because lower Likert scores indicate higher perceived severity, mean values below 3 reflect more severe or frequent psychological manifestations. Many students reported that they were “experiencing difficulties in choosing a career (believing their capabilities do not meet career requirements or lacking information about professions)”, “unable to identify a specific job in the future that aligns with their field of study”, and “feeling pressured by family, friends, or society when making career choices” (mean scores < 3) (Table 6).

#### 3.1.3. Difficulties in Adapting to the Social Environment

The survey results regarding students’ difficulties in adapting to their environment and social context indicate that many students continue to face challenges in communication and in establishing relationships with friends and particularly in their relationships with lecturers. Within family relationships, most students reported that family members generally show care and support for one another. However, many students expressed that they “find it difficult to talk comfortably or share academic matters and student life with their parents or family members” (Mean = 3.014). In peer relationships, students also reported difficulties “in initiating and maintaining close relationships with friends of the opposite sex” (Mean = 3.058). As lower Likert scores correspond to greater severity, mean values < 3 in the tables indicate more severe psychological manifestations.

More notably, within the university environment, the most serious difficulties involve academic and administrative interactions. Students indicated that they “experience difficulties when approaching, asking questions, or discussing academic or research-related matters with lecturers” and “experience difficulties when contacting or discussing personal or academic issues with university staff” (Mean < 3) (Table 7). These findings suggest that the learning environment in Vietnamese universities is not yet fully opened. Traditional hierarchy in teacher–student communication remains prevalent, and therefore the gap between students and lecturers is still considerable.

Within the university environment, Vietnamese students also encounter difficulties related to learning conditions and study methods. The most serious challenge they face is the inability to find an effective study method, followed by “difficulties in listening to lectures, remembering, and absorbing specialized knowledge” (Mean < 3) (Table 8). These difficulties can negatively affect students’ academic performance and, consequently, reduce the overall effectiveness of university education.

#### 3.1.4. Negative Adaptive Attitudes and Behaviors

The data indicate that students not only experience emotional and cognitive difficulties but also exhibit negative coping behaviors that may pose serious risks to their mental and social well-being. Consistent with the scale anchors, lower mean scores represent higher perceived severity, while higher scores indicate lower symptom severity. Many students reported that they “no longer feel interested or motivated to study as before” or “no longer feel interested in or tend to avoid participating in Youth Union/Student Association activities” (Mean < 3) (Table 9).

The use of alcohol, beer, e-cigarettes, or even stronger stimulants to relieve stress reflects a concerning trend in students’ stress-coping mechanisms. However, in this study, the use of stimulants such as alcohol, beer, e-cigarettes, or drugs to alleviate stress was almost nonexistent among students in economics and business administration (Mean > 4).

With respect to behaviors involving avoidance, harming relationships, or self-harm—such as “being easily agitated and showing violent reactions toward others”, “self-injury (cutting, hitting, or inflicting harm on oneself) to reduce distress”, or “having suicidal intentions”—these manifestations were not severe among the surveyed Vietnamese students (Mean > 3).

#### 3.1.5. Analysis Cronbach’s Alpha Reliability Test

##### Reliability Analysis (Cronbach’s Alpha)

To assess the current status of psychological issues and mental health among students in economics-related disciplines, the study employed a set of variables and measurement scales adapted from internationally validated psychological and mental health instruments.

The initial reliability analysis indicated that all scales achieved Cronbach’s alpha values above the acceptable threshold of 0.60. Item–total correlation analysis was subsequently conducted to identify items that did not meet the required correlation criteria. After this exclusion, all remaining items demonstrated corrected item–total correlations greater than 0.30 and lower than the corresponding overall Cronbach’s alpha values (see Appendix A).

All scales therefore met the reliability criteria and were deemed suitable for Exploratory Factor Analysis (EFA). The final results confirmed satisfactory internal consistency of the measurement scales and an adequate correspondence between the observed data and the proposed constructs.

##### Exploratory Factor Analysis (EFA)

Exploratory Factor Analysis (EFA) was conducted to examine the underlying factor structure of the measurement scales. The KMO value was 0.940 and Bartlett’s test of sphericity was significant (*p* < 0.001), confirming the suitability of the data for factor analysis. Principal Component Analysis with Promax rotation was applied. After removing 16 items with low or cross-factor loadings, 31 observed variables were retained and grouped into seven factors. The extracted factors had eigenvalues greater than 1 and explained 64.019% of the total variance, meeting the recommended criteria (see Appendix B). These results indicate an adequate factor structure for subsequent Confirmatory Factor Analysis (CFA).

##### Confirmatory Factor Analysis (CFA)

In this paper, the CFA was conducted to verify the adequacy and coherence of individual observed variables within their respective latent constructs. The analysis was used for construct validation purposes only; no composite or factor scores were computed, and subsequent analyses and interpretations are based on item-level results. The results of the Confirmatory Factor Analysis (CFA) indicated an acceptable model fit. The goodness-of-fit indices showed χ^2^/df = 4.155, GFI = 0.886, CFI = 0.886, and RMSEA < 0.07, suggesting that the proposed measurement model adequately fit the observed data.

Model reliability and validity were further assessed through Composite Reliability (CR), convergent validity, and discriminant validity. All constructs exhibited CR values ranging from 0.775 to 0.841, exceeding the recommended threshold of 0.70. Convergent validity was supported as all Average Variance Extracted (AVE) values were greater than 0.50. Discriminant validity was also confirmed, with MSV values lower than AVE and the square roots of AVE exceeding inter-construct correlations. (see Appendix C).

After removing items with low reliability, the final CFA model retained 16 observed variables representing five latent constructs. Overall, the CFA results confirmed the adequacy of the measurement model, which was subsequently used for hypothesis testing and further statistical analyses. In addition, the CFA results indicate full consistency between the empirically validated factor structure and the factor groupings proposed in the theoretical framework. Conceptual dimensions used to organize the needs assessment are therefore aligned with, but analytically distinct from, the statistical measurement model.

#### 3.1.6. The Interconnections Between Mental State and Psychological Difficulties

For Chi-square and odds ratio analyses, Likert-scale items were dichotomized to define exposure status. Responses indicating “very severe” or “severe” (scale points 1–2) were classified as “exposed”, representing the presence of psychological difficulties or stress-related conditions, whereas responses of “unclear”, “not severe”, or “not severe at all” (scale points 3–5) were classified as “non-exposed”. This dichotomization approach was applied consistently across predictor and outcome variables to facilitate screening-level association analysis rather than causal inference.

In examining the magnitude and direction of the associations through odds ratios, all pairs of variables were assessed using the Chi-square test and demonstrated statistically significant results (*p* = 0.001) (see Appendix D). Accordingly, the following conclusions were drawn:

Overall, the results indicate that mental health difficulties, challenges in career orientation, and negative adaptive behaviors among students are closely interrelated. Depressive symptoms and career uncertainty were associated with higher odds of difficulties across academic, daily life, and social domains, highlighting the multidimensional and interconnected nature of student mental health in the higher education context.

##### Adapting to the Academic and Social Environment

The Crosstab analysis indicated statistically significant associations between depressive symptoms (including feelings of sadness, stress, and loneliness) and difficulties in adapting to the academic environment and student life.

Specifically, students who frequently reported feelings of sadness, hopelessness, and low mood were 3.04 times more likely to report discomfort and dissatisfaction with the university environment compared to those without such symptoms (OR = 3.040; 95% CI: 2.225–4.154). These students also showed 2.74-fold higher odds of reporting difficulties in daily living during university studies and 2.81-fold higher odds of reporting pressure, regret, or distress related to independent living.

For students reporting frequent stress, pressure, and difficulty initiating tasks, the associations were stronger, with odds ratios ranging from 3.44 to 4.10. In particular, the likelihood of experiencing pressure and regret associated with independent living was 4.10 times higher among stressed students than among their non-stressed counterparts (OR = 4.102; 95% CI: 2.638–6.378).

Additionally, students who frequently felt lonely or socially isolated showed significantly higher odds of experiencing adaptation difficulties, with odds ratios ranging from 2.08 to 3.98. The strongest association in this group was observed for feelings of pressure and regret related to independent living.

##### Career Orientation and Negative Self-Directed Attitudes and Adaptive Behaviors

Further Crosstab analysis demonstrated clear associations between difficulties in career orientation and negative self-directed attitudes and adaptive behaviors among students.

Students experiencing difficulties in career choice were:

- 4.19 times more likely to report a loss of confidence in academic abilities and problem-solving skills (OR = 4.189; 95% CI: 2.865–6.124),
- 4.00 times more likely to report reduced interest and motivation in learning,
- 2.83 times more likely to report avoid participation in Youth Union and Student Association activities,
- 2.84 times more likely to report disengage from community and social activities.

Similarly, students who were unable to identify a specific future occupation aligned with their field of study demonstrated approximately a threefold likely percentage of reduced self-confidence and academic motivation, accompanied by a greater tendency toward social withdrawal. Among students who reported experiencing pressure from family, peers, or society regarding career choices, the odds of exhibiting negative adaptive behaviors ranged from 2.30 to 2.76, indicating a consistent association between external pressure and adverse behavioral outcomes.

### 3.2. Psychological and Mental Health Support Needs Among Students

#### 3.2.1. Self-Perceived Support Needs

The findings indicate that nearly 30% of students self-identify and affirm that they are in need of psychological and mental health (MH) support, while an additional 30% remain uncertain about whether they require such support. Only around 40% of students assert that they do not need psychological or MH assistance. These results suggest that students’ support needs are substantial, especially given that a large proportion remain hesitant or unclear about their own needs. It is highly likely that many of these students may be experiencing latent psychological or MH difficulties without fully recognizing them.

Despite this considerable level of need, many students experience feelings of hesitation or avoidance when discussing the idea of “seeking psychological support.” In-depth interviews show that many students have experienced stress, anxiety, insomnia, or loss of study motivation, yet do not know whom to approach or feel reluctant to share their struggles. A student from the University of Economics Ho Chi Minh City (UEH) remarked: “*There were times when I really needed someone to talk to, but I was afraid people would think I was weak*”. A male student from the School of Economics, Vinh University (VinhUni) shared: “*I know I’m stressed, but I don’t know who to ask for help*”. Other students reported that they “didn’t want to bother others” or were “afraid of being seen as weak”. These insights demonstrate that the need for psychological support is both real and widespread; however, it is often suppressed by psychological, perceptual, and cultural barriers.

#### 3.2.2. Needs for Specific Support Content

**Figure 3:**
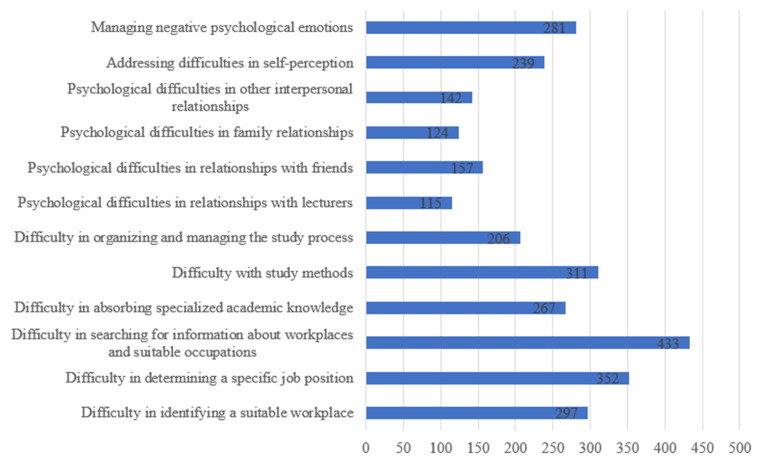
Issues for Which Students Self-Identify a Need for Support. (Source: Authors’ survey, 2025).

The survey results show that students’ perceived support needs primarily cluster into four major categories: (1) career orientation, (2) academic difficulties, (3) psychological and social relationship issues, and (4) personal development and emotional well-being. Among these, students expressing a need for career orientation support accounted for the highest proportion. This is reflected in items such as “seeking information about suitable workplaces and professions” (61.7%), “identifying job positions” (50.2%), and “identifying workplaces” (42.3%). These figures indicate that a large number of students are still in the process of exploring and defining their career pathways, underscoring an urgent need for guidance and support in planning their professional futures (Figure 3).

The second major area of need relates to support in “overcoming academic difficulties”, which also accounts for a significant proportion. Specifically, 44.3% of students wish to receive support in resolving *difficulties with study methods*, 38.0% need assistance in tackling *challenges related to absorbing specialized knowledge*, and 29.4% express a need for support in *organizing and managing their study process*. These results reflect the reality that many students still lack essential university-level study skills, including self-studying, time management, and effective approaches to mastering academic content. Students clearly express a growing need for support in addressing these learning challenges. In interviews, a student from UEH shared: “*I do study a lot, but it seems ineffective. And I don’t know where to start*”. Similarly, a student from NEU said: “*There is so much coursework, but no one tells us how to study properly; so it’s very easy to get overwhelmed and stressed*”.

The next group of support needs identified by students relates to psychological–social aspects. Many students indicated a need for support in addressing “difficulties in relationships with friends” (22.4%), “difficulties in family relationships” (17.7%), “difficulties in relationships with lecturers” (16.4%), and “other relational difficulties” (20.25%). These findings highlight that issues of communication, social isolation, and interpersonal conflict are affecting students’ mental health, and that students themselves are, to some extent, aware of these challenges and recognize their need for support.

Finally, issues related to personal development and internal emotional well-being were also perceived as areas requiring support. Specifically, 40.1% of students expressed a need for assistance in “managing negative psychological emotions”, and 34.1% reported needing “support in self-understanding”. This reflects a growing tendency among students to seek greater self-awareness, improve emotional regulation, and enhance adaptive capacity in an increasingly demanding academic and social environment.

Overall, students’ support needs are multidimensional and interrelated. Academic and career-related difficulties often trigger psychological stress, while emotional and interpersonal issues negatively impact learning motivation and future orientation.

Therefore, support for students cannot be fragmented into isolated components; rather, it must be designed as an integrated model that addresses multiple domains simultaneously—strengthening academic competence, promoting mental health, and fostering positive social skills. These findings suggest that higher education institutions should develop comprehensive psychological support and career guidance programs that enable students to achieve sustainable development in knowledge, emotional well-being, and personal identity.

#### 3.2.3. Needs Regarding Forms of Support

The quantitative survey results indicate that students wish to access a variety of support formats, with a strong preference for personalized, direct, and highly confidential forms of assistance. The findings show that individual counseling is the most commonly preferred support modality (69.6%), far surpassing other forms such as small-group counseling (18.1%), individual consultation (31.2%), or counseling websites (32.4%). This demonstrates that students prefer private conversations where they can be heard and understood in a safe environment, rather than participating in group-oriented support activities. All forms involving large groups or unclear levels of confidentiality—such as “large-group communication”, “group counseling”, or “counseling hotlines”—were generally not favored by students.

Qualitative data analysis further reveals that many students hesitate to share in front of others and feel comfortable only when trust is established on a personal level. A student from UEH stated: “*I want to talk privately with one person; I don’t want anyone else to know because I’m afraid of being judged*”. Another student from VinhUni added: “*If there were a private counseling room where I could talk to someone who understands psychology, I would be willing*”. Thus, individual counseling and private consultations are preferred not only for their perceived effectiveness but also because they provide a sense of safety and protection from stigma—an important factor given the persistent hesitation among Vietnamese students to seek psychological support.

“Counseling websites” (32.4%) were also more preferred than “counseling hotlines” (11.8%). One student shared: “*If there were a website or app where I could message someone, it would be easier. Calling or meeting directly can feel very uncomfortable*”. This suggests that developing online counseling systems, psychological chatbots, or student support websites is a promising direction—aligned with young people’s digital habits and conducive to early psychological interventions.

Additionally, formats such as “*small-group counseling*” and “*group consultations*”, although selected by a smaller proportion of students, still hold value in building peer support communities. Several interviewed students remarked that sharing in small groups helps them “*realize they are not alone*” and “*learn different perspectives on their problems*”. However, willingness to participate remains limited due to concerns about personal information disclosure and uncertainty regarding the confidentiality of group-based activities.

#### 3.2.4. Needs Regarding Support Methods

**Figure 4:**
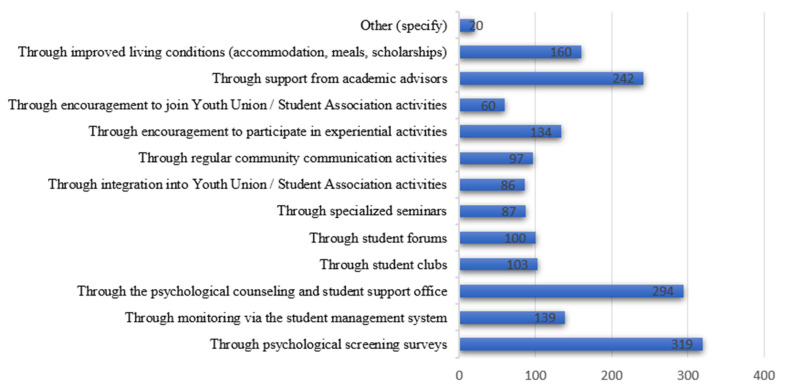
Preferred support methods. (Source: Authors’ survey, 2025).

The findings indicate that students highly value, and express a strong need for, psychological and mental health support delivered through methods integrated within the university environment—particularly those that are formal, regular, and systematic. The most frequently selected methods include “*psychological screening surveys*” (45.5%) and “*psychological counseling and student support offices*” (41.9%). These preferences reflect students’ desire for continuous monitoring and proactive care from their universities, rather than support that is only provided after a crisis has occurred (Figure 4).

Several interviewed students shared that they strongly wish for universities to proactively engage with them, conduct check-ins, or organize periodic mental health assessments, since “*many students do not know how stressed they actually are*”. This observation aligns with the finding that 30% of surveyed students remain uncertain about whether they need psychological or mental health support. A student from VinhUni commented: “*If the university had regular assessments or someone checked in on us, I would feel more cared for*”. This highlights that proactive actions and effective implementation by university student affairs and support units are key to early identification of psychological issues and to building student trust in seeking help.

Support methods embedded within academic and campus-life activities were also selected at relatively high rates. Approximately 34.5% of students expressed a desire for support from “*academic advisors*”, while 22.8% wished to receive support through “*improved living conditions (dormitories, meals, scholarships, etc*.)”. This suggests that students perceive mental health as inseparable from their learning and living environment, and that they want an integrated support network involving lecturers, academic advisors, student unions, and student affairs offices.

Notably, around 20% of students preferred being supported through “*experiential activities*” or “*encouragement to participate in youth-union/student-association activities*”, pointing to a need for indirect support channels through social interaction, community building, and positive collective engagement. In interviews, several students shared that “*participating in clubs makes them happier, more confident, and less lonely*”. This underscores the role of the university environment as an expanded psychosocial support system, rather than solely an academic institution.

#### 3.2.5. Needs Regarding Support Providers

The survey results indicate that students tend to seek help from formal, professional sources, especially individuals with expertise in psychology and mental health. The two most preferred groups are psychological counselors (53.9%) and school-based psychological or social workers (25.5%). These choices reflect students’ increasingly positive perceptions of professional mental health support and their desire to be heard and guided by trained, qualified practitioners.

**Figure 5:**
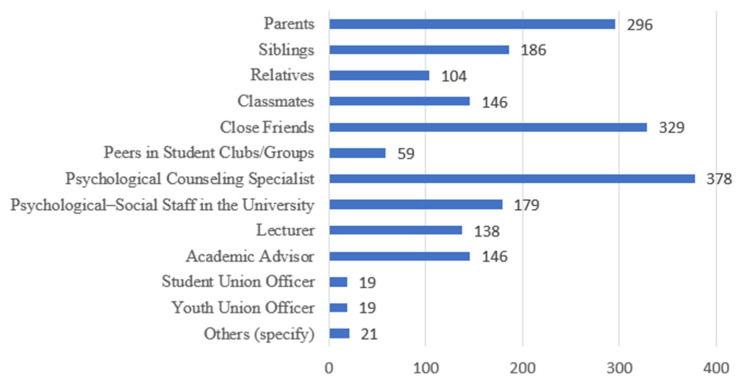
Preferred Support Providers. (Source: Authors’ survey, 2025).

Alongside professionals, close personal relationships are also seen as important sources of support. Figure 5 shows that parents are the third-most chosen group (42.2%) while close friends are the second group (46.9%) and the fourth is siblings (26.5%). This highlights the central role of family and friends in the spiritual life of Vietnamese university students—where familiar, intimate relationships remain their primary emotional anchors in times of difficulty.

However, qualitative data show that not all students find it easy to share with family members. This may stem from generational gaps that make parents less attuned to the realities of university life today, or from the fact that many students live far from home while studying in urban centers. A UEH student shared: “*I don’t tell my parents because I’m afraid they’ll worry, so I usually only talk to my close friend*”. A student from UFM expressed: “*My family is strict, so sometimes I want to talk but don’t know how to start*”. These shares reveal that although family and close friends are important support sources, these relationships also involve psychological barriers—particularly fears of judgment or misunderstanding.

A smaller group of students expressed interest in being supported by lecturers, academic advisors, or university organizations. Specifically, 20.8% of students selected academic advisors, and 19.7% selected lecturers. Notably, among 700 surveyed students, only 19 students chose Youth Union/Student Association officers as potential providers of psychological or mental health support. This extremely low percentage suggests that students do not perceive these organizations as reliable sources of mental health support, or that there is a lack of clear mechanisms connecting students with these officers. This finding suggests that the Youth Union and Student Association should re-evaluate their organizational models and activities to determine whether they are effectively fulfilling their mission of guiding, inspiring, and supporting students’ personal and aspirational development.

Several students also reported hesitation in sharing with lecturers due to fear that it might affect their academic performance, or because lecturers are perceived as “busy” or “difficult to approach.” Students still tend to view lecturers as distant authority figures. This suggests a need to strengthen lecturers’ and academic advisors’ capacity to identify early signs of student distress and to act as “bridges” connecting students with professional counseling services. It also points to the importance of raising faculty awareness about their broader role as educators—who not only deliver academic knowledge but also support, inspire, and accompany students in their development.

In addition, a small proportion of students (under 10%) reported that they prefer sharing with peers in student clubs or groups. This reflects a growing interest in peer support, an emerging trend in school-based mental health services, where students help one another through shared experiences, active listening, and empathy.

## 4. Discussion

Overall, the findings of this study provide robust evidence that psychological difficulties among Vietnamese university students majoring in economics are widespread, multidimensional, and strongly intertwined with perceived needs for psychological and mental health support. Extending existing international and Vietnamese research, the findings demonstrate that mental health challenges in this population are not limited to isolated emotional symptoms, but rather emerge from the interaction between academic demands, career uncertainty, social adaptation, and individual coping capacity.

Consistent with international literature, negative emotional states—particularly anxiety, stress, and depressive symptoms—emerged as the most common psychological difficulties among students. Frequent worries about time management, financial conditions, family circumstances, and physical health reflect an imbalance between environmental demands and students’ perceived control over their academic and personal lives.

This pattern aligns with the Demand–Control Model, which posits that high demands combined with limited perceived control increase psychological strain. The strong associations observed between depressive symptoms and difficulties in academic adaptation, daily living, and independent life further support the multidimensional view of student mental health, as conceptualized in OECD [35] and WHO [13] frameworks, where emotional distress may cascade into cognitive, social, and behavioral dysfunctions.

A key contribution of this study lies in identifying career orientation difficulties as a central stressor shaping mental health outcomes among economics students. Uncertainty regarding future employment, perceived mismatches between personal competencies and labor-market expectations, and intense pressure from family and society were strongly associated with reduced self-confidence, declining learning motivation, and social withdrawal. While previous Vietnamese studies have noted career anxiety as a common concern, this study empirically demonstrates that career-related stress functions as a core mechanism linking emotional distress to maladaptive attitudes and behaviors [23,50]. From the perspective of basic psychological needs theory, these findings indicate unmet needs for competence and autonomy, particularly when students perceive limited agency in career decision-making or experience externally imposed expectations. This interpretation is consistent with Nguyen Thi Mai Huong [17], who emphasized the role of reduced self-efficacy and constrained autonomy in undermining students’ emotional well-being. Compared with international studies that primarily emphasize academic workload or financial stress, the prominence of career orientation difficulties in this study reflects the specific socio-cultural and labor-market context of Vietnam, where higher education is closely tied to expectations of stable employment and upward social mobility [15,25].

The findings on social adaptation further highlight structural and cultural characteristics of Vietnamese higher education. Difficulties in communicating with lecturers and university staff were more pronounced than problems within family or peer relationships. This suggests that hierarchical teacher–student dynamics and limited psychosocial interaction within academic institutions may act as barriers to informal help-seeking. Such dynamics help explain why students expressed a strong preference for professional, confidential counseling services rather than seeking support from lecturers, student organizations, or administrative units. This finding is consistent with prior Vietnamese studies; however, this pattern contrasts with Western higher education contexts, where faculty members often play a mentoring and advisory role in students’ personal and academic development. Such structural and cultural factors help explain students’ preference for professional counselors and confidential individual support rather than seeking help from lecturers or student organizations.

Regarding support needs, a substantial proportion of students either explicitly reported a need for psychological or MH support or remained uncertain, suggesting the presence of latent distress and limited mental health awareness. This pattern is consistent with Vietnamese studies indicating that students often experience psychological difficulties but hesitate to label them as mental health problems or seek formal assistance [47,56]. Students tended to articulate their needs in functional and developmental terms—such as career guidance, learning strategies, emotional regulation, and self-understanding—rather than in clinical language. This finding is consistent with previous Vietnamese research indicating that students often normalize psychological distress and prioritize practical coping over formal mental health labeling [15]. The strong preference for individual and confidential counseling reflects persistent stigma and fear of social judgment, as previously noted by Nguyen Thi Minh Hang [44] and Tran Thi Thu Ha et al. [18]. Meanwhile, emerging interest in online counseling aligns with recent recommendations by UNICEF [12] and WHO [13] to expand low-threshold, digitally mediated mental health interventions for young people.

Overall, the discussion highlights that student mental health cannot be effectively addressed through fragmented or purely reactive interventions. Psychological difficulties, academic challenges, and career uncertainty form a tightly interwoven system that requires integrated, preventive, and context-sensitive support models within universities.

Several strengths of this study should be noted. The research is grounded in a clear theoretical framework, employs reliable and validated measurement scales, and combines quantitative and qualitative data to capture both prevalence and lived experiences. Focusing on economics students addresses a notable gap in the Vietnamese literature and provides discipline-specific evidence for targeted policy and practice. However, limitations include the cross-sectional design, reliance on self-reported data, and potential constraints in generalizing findings across all institutional and regional contexts in Vietnam.

Future research should adopt longitudinal and intervention-based designs to examine causal mechanisms and evaluate the effectiveness of integrated support models that combine career guidance, academic skills development, and psychological counseling. Comparative studies across academic disciplines would also help clarify whether the observed patterns are unique to economics students or reflect broader systemic challenges in Vietnamese higher education.

## 5. Conclusions

This study examined psychological difficulties and mental health support needs among Vietnamese university students majoring in economics using a mixed-methods, need-based approach. The findings indicate that student mental health challenges are prevalent and multidimensional, extending beyond isolated emotional symptoms to include cognitive difficulties, maladaptive behaviors, and challenges in academic and social adaptation. Mental health risks among economics students do not primarily manifest as severe psychiatric disorders, but rather as persistent anxiety, prolonged stress, sleep and eating disturbances, declining academic motivation, and uncertainty about future orientation. These patterns suggest that a substantial proportion of students remain in a psychologically vulnerable state that may intensify without timely and appropriate support.

A central contribution of the study is its discipline-specific focus, demonstrating that career orientation difficulties play a pivotal role in shaping students’ emotional well-being and adaptive behaviors. Perceived skill mismatches, unclear career pathways, and strong family and societal pressure were closely associated with reduced confidence, lower learning motivation, and social withdrawal. Students articulated their support needs mainly in developmental and functional domains, including career guidance, academic learning strategies, emotional regulation, and self-understanding, and showed a strong preference for individualized, confidential, and professionally delivered support. These findings extend existing literature by showing that career-related concerns function not merely as contextual stressors, but as core mechanisms shaping students’ mental health and adaptive behaviors.

Conceptually, this research advances a need-based perspective on student mental health by shifting attention from symptom prevalence to students’ perceived support needs. Students primarily articulated their needs around functional and developmental domains, including career guidance, academic learning strategies, emotional regulation, self-understanding, and interpersonal problem-solving. This suggests that students do not predominantly interpret their difficulties as clinical “mental illness”, but rather as challenges in coping with academic demands, future uncertainty, and social relationships. The strong preference for individualized, confidential, and professionally delivered support further reflects persistent concerns about stigma and privacy within the Vietnamese context.

The findings of this study underscore the need for integrated and preventive mental health support systems in higher education that combine early screening, accessible professional counseling (including digital formats), and close integration with academic advising and career services. Addressing these challenges requires a multidimensional, need-based approach that aligns institutional support systems with students’ academic and developmental realities. By offering discipline-sensitive empirical evidence, this study contributes to the development of more responsive and sustainable mental health support models in Vietnamese universities and comparable educational contexts.

## Figures and Tables

**Table 1:** Negative and pathological psychological dimension scale (PD).

Factor	Reference	Variables	Questions
PD1: Anxiety disorder	GAD7; DASS-21	- PD11. Anxiety/excessive worry: Living conditions in the university environment - PD12. Anxiety related to time management and organization of daily activities - PD13. Anxiety related to financial and economic conditions - PD14. Anxiety related to current family circumstances - PD15. Anxiety related to personal health, physical appearance, and related concerns	V-pd11: You frequently feel worried about living conditions at the university (including classrooms, dormitories, and the overall university environment)? V-pd12: You frequently feel worried about managing your time and organizing daily activities (e.g., class schedules, self-study, personal routines, and collective activities)? V-pd13: You frequently feel worried about your personal financial situation (including tuition fees, living expenses, and other unforeseen costs). V-pd14: You frequently feel worried about your current family circumstances (e.g., family members’ health and household economic conditions). V-pd15: You frequently feel worried about your personal health or physical appearance (e.g., physical health, body weight, and perceived attractiveness).

**Table 2:** Distribution of the survey sample.

School Year University	First Year	Second Year	Third Year	Fouth Year	Total
NEU	37	81	42	11	171
FTU	123	38	40	13	214
VinhUni	6	63	26	11	106
DNU	17	6	13	1	37
UEH	103	70	0	0	173
Total	286	258	121	36	701

(Source: Authors’ survey, 2025).

**Table 3:** Self-perceived manifestations of anxiety disorders.

Observed Variable	Mean	Median	Mode
Frequently feeling anxious about living conditions at university (including classrooms, dormitories, and the overall campus environment)	3.097	3	4
Frequently feeling anxious about managing time and organizing daily activities (such as scheduling classes, self-study, personal routines, and group activities)	2.234	2	2
Frequently feeling anxious about one’s personal financial situation (including tuition fees, living expenses, and unexpected costs)	2.094	2	2
Frequently feeling anxious about current family circumstances (such as family members’ health and household economic conditions)	2.255	2	2
Frequently feeling anxious about personal health or physical appearance (physical health, body weight, attractiveness)	2.083	2	2

(Source: Authors’ survey, 2025).

**Table 4:** Self-perceived manifestations of depression.

Observed Variable	Mean	Median	Mode
Frequently feeling bored, disappointed, or downhearted	2.820	3	2
Frequently feeling stressed, pressured, and having difficulty initiating tasks	2.653	3	2
Frequently feeling lonely or isolated at school, in class, or within the family, even when surrounded by friends or relatives	3.177	3	4

(Source: Authors’ survey, 2025).

**Table 5:** Self-perceived manifestations of behavioral and psychotic disturbances.

Observed Variable	Mean	Median	Mode
Frequently feeling easily agitated, irritable, or losing control over one’s behavior	3.257	3	4
Frequently displaying aggressive, impulsive behaviors or acting without considering consequences in academic or life decisions	3.787	4	4
Frequently experiencing disorganized thoughts, exaggerated thinking, strange ideas, or obsessions	3.190	3	4
Believing that one can easily achieve all goals in study and life	3.220	3	3
Frequently experiencing loss of appetite or overeating	2.820	3	2
Frequently experiencing insomnia, lack of sleep, difficulty falling asleep, poor sleep quality, or oversleeping	2.772	2	2

(Source: Authors’ survey, 2025).

**Table 6:** Perceptions of difficulties in self-understanding and career orientation.

Observed Variable	Mean	Median	Mode
Feeling not being appreciated or valued by people around	3.471	4	4
Perceiving themselves as having nothing to be proud of and considering themselves a failure	3.414	4	4
Feeling difficulties in choosing a career (believing personal capabilities do not meet job requirements, lacking information about occupations)	2.324	2	2
Being unable to identify a specific future job that aligns with their current field of study	2.471	2	2
Feeling pressured by family, friends, or society when making career choices	2.810	3	2

(Source: Authors’ survey, 2025).

**Table 7:** Perceptions of difficulties in relationships with family, friends, and teachers.

Observed Variable	Mean	Median	Mode
Finding it difficult to talk comfortably or share academic matters and student life with parents or family members	3.014	3	4
Feeling that parents or family members do not understand, frequently disagree, or often experience conflicts	3.354	4	4
Feeling a lack of care and support within family	3.916	4	4
Finding it difficult to communicate, express thoughts, and convey emotions to friends	3.211	3	4
Feeling unable to well integrate in group activities (feeling isolated or not belonging to the group)	3.314	3	4
Experiencing difficulties in initiating and maintaining close relationships with friends of the opposite sex	3.058	3	4
Experiencing difficulties when approaching, asking questions, or discussing academic and research matters with lecturers	2.943	3	2
Experiencing difficulties when communicating with university staff about personal or academic issues	2.892	3	2

(Source: Authors’ survey, 2025).

**Table 8:** Perceptions of difficulties in adapting to the university learning environment.

Observed Variable	Mean	Median	Mode
Frequently feeling discomfort or frustration with the university environment (class schedule, classroom space, library, campus landscape, sanitation conditions, etc.)	3.348	4	4
Frequently feeling discomfort or frustration with daily life while studying at university (rented accommodation, dormitories, commuting, etc.)	3.214	3	4
Feeling pressured, regretful, or even remorseful due to having to be more independent as a university student	3.578	4	4
Experiencing difficulties in listening to lectures, remembering, and absorbing specialized knowledge	2.934	3	2
Still struggling to find an effective study method	2.441	2	2
Frequently losing control over study tasks (e.g., mixing up class schedules, missing assignment deadlines, etc.)	3.635	4	4

(Source: Authors’ survey, 2025).

**Table 9:** Perceptions of losing confidence and losing motivation.

Observed Variable	Mean	Median	Mode
Feeling a loss of confidence in learning abilities and problem-solving skills	3.040	3	4
Feeling no longer interested or motivated to study as before	2.907	3	2
No longer interested in or avoiding participation in Youth Union/Student Association activities	2.924	3	2
No longer caring about engaging in community or social activities (birthdays, events, volunteering, or social gatherings with friends)	3.332	4	4

(Source: Authors’ survey, 2025).

**Figure A1:**
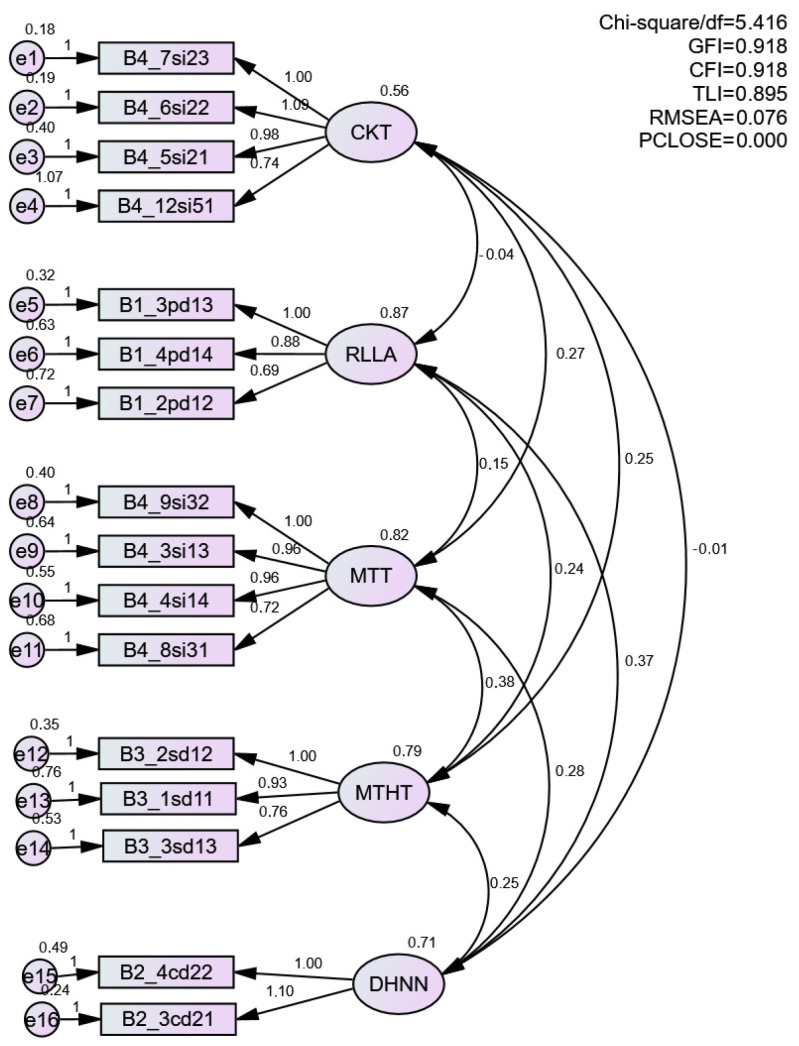
Final standardized CFA results.

## Data Availability

The original contributions presented in this study are included in the article. Further inquiries can be directed to the corresponding author.

## Appendix A

**Table A1:** Results of Cronbach’s Alpha test for the factors.

	Scale Mean if Item Deleted	Scale Variance if Item Deleted	Corrected Item-Total Correlation	Cronbach’s Alpha if Item Deleted
B1_1 (pd11). You are frequently worried about living conditions at school (including classrooms, dormitories, and the university environment).	32.901	74.572	0.444	0.868
B1_2 (pd12). You often worry about managing your time and organizing daily activities (scheduling classes, self-study; personal activities, group activities, etc.).	33.740	75.084	0.478	0.866
B1_3 (pd13). You are frequently worried about your financial situation (including tuition fees, living expenses, and other unforeseen costs).	33.899	73.928	0.528	0.863
B1_4 (pd14). You are frequently worried about your current family situation (health of family members, household economic conditions).	33.733	73.452	0.522	0.863
B1_5 (pd15). You frequently worry about your personal health or appearance (physical health, weight, appearance).	33.895	75.820	0.479	0.865
B1_6 (pd21). You often feel depressed, disappointed, and downcast.	33.142	70.932	0.676	0.854
B1_7 (pd22). You often feel stressed, pressured, and find it difficult to start any task.	33.314	71.268	0.676	0.855
B1_8 (pd23). You often feel lonely and isolated at school, in class, and at home, even when you are with friends and family.	32.798	71.333	0.600	0.859
B1_9 (pd31). You often feel easily agitated, angry, and lose control of your behavior.	32.721	72.167	0.572	0.860
B1_10 (pd32). You tend to act aggressively, impulsively, and without considering the consequences in your academic and life decisions.	32.181	75.417	0.476	0.866
B1_11 (pd41). You find yourself thinking in a chaotic, excessive way, having strange or obsessive thoughts.	32.791	71.029	0.621	0.857
B1_13 (pd51). You often experience loss of appetite or excessive cravings.	33.152	73.638	0.508	0.864
B1_14 (pd52). You frequently experience insomnia, sleep deprivation, difficulty falling asleep, shallow sleep, or excessive sleep.	33.198	73.301	0.492	0.865
B2_1 (cd11). You notice that the people around you never appreciate you.	11.080	10.647	0.419	0.730
B2_2 (cd12). You perceive yourself as having nothing to be proud of and as a failure.	11.113	9562	0.478	0.712
B2_3 (cd21). Are you having difficulty choosing a career (your abilities don’t meet the job requirements, you lack information about different professions)?	12.183	9224	0.608	0.662
B2_4 (cd22). You cannot identify any specific future job that is suitable for your field of study.	12.063	9223	0.563	0.678
B2_5 (cd23). You feel pressure from family, friends, or society when choosing a career.	11.731	9548	0.481	0.710
B3_1 (sd11). You find it difficult to talk comfortably, to share about your studies and student life with your parents and family members.	41.746	80.786	0.545	0.877
B3_2 (sd12). You feel that your parents and family members don’t understand you, that you always disagree with them, and that there are frequent conflicts.	41.389	82.470	0.537	0.877
B3_3 (sd13). You see a lack of care and support within your family.	40.831	84.565	0.465	0.880
B3_4 (sd21). You find it difficult to talk, to express your thoughts and feelings to your friends around you.	41.540	81.254	0.589	0.874
B3_5 (sd22). You find it difficult to fit in with others in group activities (you feel out of place, not belonging to the group).	41.452	80.544	0.614	0.873
B3_6 (sd23). You have difficulty initiating and maintaining close relationships with people of the opposite sex.	41.733	81.420	0.511	0.878
B3_7 (sd31). You find it difficult to interact with, ask questions about, or discuss academic and research-related issues with your teachers at school.	41.819	79.628	0.667	0.870
B3_8 (sd32). You find it difficult to communicate with school staff about personal or academic issues.	41.868	80.400	0.639	0.872
B3_9 (sd41). You experience many annoyances and frustrations with the university environment (timetable, classroom space, library, landscape, sanitary conditions, etc.).	41.414	82.061	0.574	0.875
B3_10 (sd42). You experience many annoyances and frustrations with daily life while studying at university (accommodation, dormitories, transportation, etc.).	41.555	81.493	0.571	0.875
B3_11 (sd43). You feel pressure, regret, or even remorse for having to be more independent as a student.	41.187	82.332	0.541	0.877
B3_12 (sd51). You have difficulty listening to lectures, memorizing, and absorbing specialized knowledge.	41.831	81.726	0.565	0.875
B3_13 (sd52). Are you still struggling to find an effective study method?	42.316	83.238	0.483	0.879
B3_14 (sd53). You often lose control in your studies (frequently mix up class schedules; miss deadlines for assignments, reports, etc.)	41.116	83.285	0.483	0.879
B4_1 (si11). You feel a lack of confidence in your learning and problem-solving abilities.	46.198	66.813	0.470	0.866
B4_2 (si12). You no longer feel interested in or motivated to study as before.	46.303	66.029	0.487	0.865
B4_3 (si13). You are no longer interested in or avoid participating in school’s Youth Union/Student Association activities.	46.289	65.372	0.505	0.864
B4_4 (si14). You are no longer interested in participating in community or social activities (birthdays, events, volunteering, or socializing with friends).	45.899	64.432	0.584	0.859
B4_5 (si21). You frequently drink alcohol/beer to relax or relieve stress.	44.847	67.143	0.529	0.862
B4_6 (si22). You frequently use cigarettes or e-cigarettes (vape, pod) to relax or relieve stress.	44.668	67.262	0.550	0.861
B4_7 (si23). Have you ever used stimulants (marijuana, heroin, ecstasy, etc.) to relax or relieve stress?	44.599	67.771	0.561	0.861
B4_8 (si31). You don’t want to make friends/get to know new people.	45.461	65.465	0.581	0.859
B4_9 (si32). You do not want to participate in club/group activities.	45.638	64.189	0.625	0.857
B4_10 (si41). You tend to raise your voice or say hurtful things, or mock others.	45.258	66.028	0.565	0.860
B4_11 (si42). You are easily agitated and tend to react violently towards others.	45.066	65.824	0.618	0.858
B4_12 (si51). Have you ever hurt yourself (cutting your skin, cutting your flesh, self-harming, inflicting injuries on yourself) to relieve suffering or stress?	45.128	64.795	0.542	0.862
B4_13 (si52). Have you ever had suicidal thoughts/attempted suicide?	45.303	64.649	0.502	0.865

**Table A2:** Results of Cronbach’s Alpha test for each factor.

Factor	CA Coefficient	Item-Total Correlation Coefficient	Number of Variables
B1	0.871	0.444–0.676	0/13
B2	0.744	0.419–0.608	0/5
B3	0.884	0.465–0.667	0/14
B4	0.871	0.47–0.625	0/13

## Appendix B

**Table A3:** Results of the KMO test for independent variables.

Kaiser–Meyer–Olkin Measure of Sampling Adequacy.		0.880
Bartlett’s Test of Sphericity	Approx. Chi-Square	8,649,600
	df	351
	Sig.	0.001

**Table A4:** Results Exploratory Factor Analysis (EFA) Rotated Component Matrix.

	Component						
	1	2	3	4	5	6	7
B4_7si23	0.872						
B4_6si22	0.863						
B4_5si21	0.813						
B4_12si51	0.492						
B1_3pd13		0.814					
B1_4pd14		0.765					
B1_1pd11		0.676					
B1_2pd12		0.673					
B1_5pd15		0.553					
B4_9si32			0.817				
B4_3si13			0.757				
B4_4si14			0.747				
B4_8si31			0.648				
B3_9sd41				0.719			
B3_11sd43				0.682			
B3_10sd42				0.655			
B3_6sd23				0.520			
B1_14pd52					0.761		
B1_13pd51					0.719		
B1_6pd21					0.641		
B1_7pd22					0.576		
B3_2sd12						0.800	
B3_1sd11						0.753	
B3_3sd13						0.626	
B3_4sd21						0.425	
B2_4cd22							0.861
B2_3cd21							0.824

**Table A5:** Observed variables after extraction.

Representative Factor	Symbol	Observed Variable
Anxiety disorders	RLLA	B1_1; B1_2; B1_3; B1_4; B1_5
Depression & Disorders physiologic	TC	B1_6; B1_7; B1_13; B1_14
Career guidance	DHNN	B2_3; B2_4
Family relationships	MTHT	B3_1; B3_2; B3_3; B3_4
School relations	QHNT	B3_6; B3_9; B3_10; B3_11
Lack of confidence & Avoiding relationships	MTT	B4_3; B4_4; B4_8; B4_9
Substance Use & Self-Harm	CKT	B4_5; B4_6; B4_7; B4_12

## Appendix C

**Table A6:** Model Fit CFA Assessment Index.

	Index	Evaluate
CMIN/DF	4155	Acceptable (<5)
GFI	0.886	Acceptable (>0.8)
CFI	0.886	Acceptable (>0.8)
RMSEA	0.065	Good model (<0.07)

**Table A7:** Model Validity Measures.

	CR	AVE	Student ID	MaxR(H)	CKT	RLLA	MTT	MTHT	DHNN
CKT	0.841	0.581	0.159	0.890	**0.762**				
RLLA	0.775	0.539	0.221	0.815	−0.064	**0.734**			
MTT	0.826	0.545	0.220	0,840	0.399 ***	0.176 ***	**0.738**		
MTHT	0.779	0.543	0.220	0.801	0.376 ***	0.289 ***	0.469 ***	**0.737**	
DHNN	0.813	0.686	0.221	0.834	−0.010	0.470 ***	0.370 ***	0.338 ***	**0.828**

bold represents significant values, *** *p* < 0.05.

## Appendix D

**Table A8:** Case Processing Summary.

	Cases					
	Valid		Missing		Total	
	N	Percent	N	Percent	N	Percent
“B1_6 (pd21). You often feel depressed, disappointed, and downcast. B3_9 (sd41). You experience many annoyances and frustrations with the university environment (timetable, classroom space, library, landscape, sanitary conditions, etc.).”	701	100.0%	0	0.0%	701	100.0%

**Table A9:** Crosstabulation.

		B3_9 (sd41). You Experience Many Annoyances and Frustrations with the University Environment (Timetable, Classroom Space, Library, Landscape, Sanitary Conditions, etc.).		Total
		Correct	Incorrect	
B1_6 (pd21). You often feel depressed, disappointed, and downcast.	Correct	278	121	399
	Incorrect	130	172	302
Total			408	293

**Table A10:** Chi-Square Tests.

	Value	df	Asymp. Sig. (2-Sided)	Exact Sig. (2-Sided)	Exact Sig. (1-Sided)
Pearson Chi-Square	50,100a	1	0.001		
Continuity Correction	49,012	1	0.001		
Likelihood Ratio	50,387	1	0.001		
Fisher’s Exact Test				0.001	0.001
Linear-by-Linear Association	50,029	1	0.001		
N of Valid Cases	701				

**Table A11:** Risk Estimate.

	Value	95% Confidence Interval	
		Lower	Upper
Odds Ratio for B1_6 (pd21). You often feel depressed, disappointed, and downcast. (Correct/Incorrect)	3040	2225	4154
“For cohort B3_9 (sd41). You experience many annoyances and frustrations with the university environment (timetable, classroom space, library, landscape, sanitary conditions, etc.). =Correct”	1619	1400	1871
“For cohort B3_9 (sd41). You experience many annoyances and frustrations with the university environment (timetable, classroom space, library, landscape, sanitary conditions, etc.). =Incorrect”	0.532	0.446	0.636
N of Valid Cases	701		
